# Associations Between Autistic Traits, Catastrophizing, Sensory Sensitivity, and Panic Attack Severity in Patients With Panic Disorder

**DOI:** 10.1192/j.eurpsy.2026.10646

**Published:** 2026-07-31

**Authors:** N. N. Türkel, E. Emekli, O. Ak

**Affiliations:** Psychiatry, Eskişehir City Hospital, Eskişehir, Türkiye

## Abstract

**Introduction:**

Panic disorder is characterized by recurrent panic attacks and heightened physiological arousal. Recent research has emphasized the associations between autistic traits and anxiety disorders, with evidence that individuals with panic disorder exhibit higher levels of autistic traits compared to healthy controls. However, the mechanisms linking autistic traits to panic severity remain insufficiently understood.

**Objectives:**

This study aimed to investigate the relationships between autistic traits, sensory sensitivity, catastrophizing, and panic severity in patients with panic disorder, and to test the potential mediating roles of sensory sensitivity and catastrophizing.

**Methods:**

A total of 103 patients diagnosed with panic disorder participated in the study. Participants completed the Autism-Spectrum Quotient (AQ), the Sensory Sensitivity Scale, a catastrophizing subscale, and the Panic Disorder Severity Scale (PDSS). Data were analyzed using SPSS 21.0 and PROCESS macro (Model 6) with 5,000 bootstrap resamples. Correlation analyses and serial mediation models were tested.

**Results:**

Patients with higher autistic traits reported significantly greater panic severity,and catastrophizing tendencies. Additionally, participants with high autistic traits showed increased sensory sensitivity across all sensory modalities. Panic severity was positively correlated with both autistic traits (r= 0.236, p= 0.017) and sensory sensitivity (r= 0.384, p<0.001). Serial mediation analysis revealed that the total effect of autistic traits on panic severity was significant (b = 0.41, p = 0.02). Importantly, the serial pathway AQ → sensory sensitivity → catastrophizing → panic severity was significant (b = 0.09, 95% CI [0.02, 0.19]).

**Conclusions:**

The findings indicate that the relationship between autistic traits and panic severity operates indirectly through increased sensory sensitivity and maladaptive cognitive appraisals. Sensory sensitivity appears to heighten catastrophizing tendencies, which in turn elevate panic severity. These results highlight the importance of considering autistic traits and sensory processing vulnerabilities in the assessment and treatment of panic disorder.

**Disclosure of Interest:**

None Declared

